# In patients eligible for meniscal surgery who first receive physical therapy, multivariable prognostic models cannot predict who will eventually undergo surgery

**DOI:** 10.1007/s00167-021-06468-0

**Published:** 2021-02-07

**Authors:** Julia C. A. Noorduyn, M. M. H. Teuwen, V. A. van de Graaf, N. W. Willigenburg, M. Schavemaker, R. van Dijk, G. G. M. Scholten-Peeters, M. W. Heymans, M. W. Coppieters, R. W. Poolman, the ESCAPE Research Group, V. A. B. Scholtes, E. L. A. R. Mutsaerts, J. Wolkenfelt, M. Krijnen, D. F. P. van Deurzen, D. J. F. Moojen, C. H. Bloembergen, Gast de Gast, T. Snijders, J. J. Halma, D. B. F. Saris, N. Wolterbeek, C. Neeter, D. M. M. J. Kerkhoffs, R. W. Peters, I. C. J. B. van den Brand, S. de Vos-Jakobs, A. B. Spoor, T. Gosens, W. Rezaie, D. J. Hofstee, B. J. Burger, D. Haverkamp, A. M. J. S. Vervest, T. A. van Rheenen, A. E. Wijsbek, E. R. A. van Arkel, B. J. W. Thomassen, S. Sprague, B. W. J. Mol, M. W. van Tulder, J. van der Kraan

**Affiliations:** 1 Department of Orthopaedic Surgery, Joint Research, OLVG Amsterdam, Oosterpark 9, 1091 AC Amsterdam, The Netherlands; 2 Department of Orthopaedics, Leiden University Medical Centre, Leiden, The Netherlands; 3 Department of Radiology, Dijklander Ziekenhuis, Hoorn, The Netherlands; 4 Department of Radiology, Isala, Zwolle, The Netherlands; 5 Faculty of Behavioural and Movement Sciences, Department of Human Movement Sciences, Amsterdam Movement Sciences, Vrije Universiteit Amsterdam, Amsterdam, The Netherlands; 6 Department of Epidemiology and Biostatistics, VU University Medical Centre, Amsterdam, The Netherlands; 7 Menzies Health Institute Queensland, Griffith University, Brisbane & Gold Coast, Australia; 8 Department of Orthopeadic surgery, Antonius ziekenhuis, Nieuwegein, The Netherlands

**Keywords:** Meniscus, Physical therapy, Prognostic model, Knee

## Abstract

**Purpose:**

Although physical therapy is the recommended treatment in patients over 45 years old with a degenerative meniscal tear, 24% still opt for meniscal surgery.

The aim was to identify those patients with a degenerative meniscal tear who will undergo surgery following physical therapy.

**Methods:**

The data for this study were generated in the physical therapy arm of the ESCAPE trial, a randomized clinical trial investigating the effectiveness of surgery versus physical therapy in patients of 45–70 years old, with a degenerative meniscal tear. At 6 and 24 months patients were divided into two groups: those who did not undergo surgery, and those who did undergo surgery. Two multivariable prognostic models were developed using candidate predictors that were selected from the list of the patients’ baseline variables. A multivariable logistic regression analysis was performed with backward Wald selection and a cut-off of *p* < 0.157. For both models the performance was assessed and corrected for the models’ optimism through an internal validation using bootstrapping technique with 500 repetitions.

**Results:**

At 6 months, 32/153 patients (20.9%) underwent meniscal surgery following physical therapy. Based on the multivariable regression analysis, patients were more likely to opt for meniscal surgery within 6 months when they had worse knee function, lower education level and a better general physical health status at baseline. At 24 months, 43/153 patients (28.1%) underwent meniscal surgery following physical therapy. Patients were more likely to opt for meniscal surgery within 24 months when they had worse knee function and a lower level of education at baseline at baseline. Both models had a low explained variance (16 and 11%, respectively) and an insufficient predictive accuracy.

**Conclusion:**

Not all patients with degenerative meniscal tears experience beneficial results following physical therapy. The non-responders to physical therapy could not accurately be predicted by our prognostic models.

**Level of evidence:**

III.

**Supplementary Information:**

The online version contains supplementary material available at 10.1007/s00167-021-06468-0.

## Introduction

Current guidelines state that physical therapy is the preferred first-line treatment in patients over 45 years of age with a degenerative meniscal tear [15, 19]. These guidelines are based on several randomized clinical trials (RCT) which demonstrated no clinically superiority of meniscal surgery over physical therapy in this population [2, 4–7, 9, 12, 16, 17, 21, 25]. However, not all patients experience beneficial results from physical therapy. An average of 24% (between 1.9 and 36%) of patients randomized to physical therapy still opt for meniscal surgery following conservative management [1].

Little information is available to predict at baseline the outcome of physical therapy in patients with a meniscal tear in both primary and secondary care, when a patient is referred by a general practitioner to an orthopedic surgeon. In secondary care, the patient and orthopedic surgeon may choose for surgical management, or to start a physical therapist-led exercise program. Patients rely on the orthopedics surgeons’ expertise to help decide on their treatment pathway. However, recent research showed that orthopedic surgeons were not able to predict whether patients would benefit from either meniscal surgery or physical therapy [20].

Patients with shorter symptom duration and more knee pain at baseline are more likely to undergo meniscal surgery following physical therapy [8]. However, this study did not report the accuracy of the association model. Also, the potential predictors with a continuous outcome were dichotomized before the logistic regression analysis. This makes it difficult to reliably predict which patients will undergo surgery following physical therapy based on the current literature.

Therefore, the aims of this study were to develop and internally validate multivariable clinical prognostic models to identify those patients who will undergo surgery following physical therapy.

## Materials and methods

Two prediction models were developed and internally validated for the outcome: meniscal surgery at 6 and 24 months after initial physical therapy in middle aged and older patients with a symptomatic degenerative meniscal tear. The data for this study were generated in the physical therapy arm of the ESCAPE trial. The ESCAPE trial was a multi-centre RCT comparing meniscal surgery with physical therapy in patients over 45 years old with a degenerative meniscal tear [21]. The Medical Research Ethics Committees (MEC-U; NL44188.100.13) approved the ESCAPE trial. The trial was registered at clinincaltrials.gov (NCT01850719) and The Netherlands Trial Register (NTR3908). The current study was reported according to the transparent reporting of a multivariable prediction model for individual prognosis or diagnosis (TRIPOD) statement [11].

### Participants

Patients aged between 45 and 70 years were referred by a general practitioner to the orthopaedic surgeon for diagnosis and treatment of their knee symptoms. All patients were diagnosed with a symptomatic degenerative meniscal tear. Besides that patients presented with symptoms, such as pain, the meniscal tear was confirmed on MRI. All patients were eligible for surgery and conservative treatment under the existing guidelines at the time. Patients who experienced a locked knee were excluded since this is an indication for surgery. In the ESCAPE-trial, patients were randomized to either immediate surgery or physical therapy. The physical therapy program consisted of a physical therapist-led standardized incremental exercise program containing of coordination/balance, closed kinetic chain strengths and cardiovascular exercises (see Appendix 1). The program was designed for 8 weeks with a total of 16 treatment sessions, each with a duration of 30 min [21]. As the Dutch health insurance does not cover PT, all 16 sessions were reimbursed by our research grant. If knee symptoms persisted following the physical therapy program (e.g., knee pain, limitations in daily activities or mechanical dysfunction), additional physical therapy sessions could be attended (not reimbursed by the study) or meniscal surgery, depending on a shared decision after consultation with the orthopaedic surgeon. All participants provided written informed consent [22].

### Outcome

The outcome was opting for meniscal surgery following physical therapy. Patients who attended less than six physical therapy sessions were excluded for the analyses. At both the 6 months and the 24 months follow up, the binary outcome was whether patients who were randomized to physical therapy treatment had undergone delayed surgery (1) or not (0).

### Candidate predictor selection

From an extensive list of baseline variables assessed within the ESCAPE trial, candidate predictors were selected using a combination of three methods. First, a literature search was conducted to identify factors associated with the outcome after physical therapy treatment in patients with a meniscal tear. The search strategy can be found in Appendix 2. Second, an electronic survey was sent to an expert panel of orthopaedic surgeons (*N* = 24), physical therapists (*N* = 22) and patients (*N* = 10) who were involved in the ESCAPE trial to identify the most relevant prognostic factors for physical therapy according to their opinion. The survey consisted of an extensive list of baseline variables assessed within the ESCAPE trial. Third, a univariable logistic regression analysis was conducted to include additional potential predictors in the prognostic models.

The selection of potential predictors contained of patients’ demographics, patient reported outcome (PROM) measures and radiographic information on MRI and radiograph. Demographic information included age, sex, level of education and body mass index (BMI). PROMs consisted of the International Knee Documentation Committee Subjective Knee Form for knee function, the visual analogue scale for pain during activities, the RAND-36 physical component scale for general physical health and patients’ expectation on pain relieve with physical therapy at 6 months following physical therapy on a 7-point Likert scale, ranging from pain will be severely worse (1) to pain will be relieved completely (7). The radiographic information consisted of the Kellgren–Lawrence score for osteoarthritis, determined on a standing radiograph in posterior–anterior direction. The information on MRI consisted of the tear location (medial, lateral or both) and the tear type according to the International Society of Arthroscopy, Knee Surgery and Orthopaedic Sports (ISAKOS) (longitudinal vertical, horizontal, radial, vertical flap, complex/degenerative, not able to classify).

A final selection of 10 potential predictors was made by the principle researchers of this study (JCAN, VAG, NWW and RWP). Then the potential predictors were ranked to decide which will be included in the model, based on the 10 events per potential predictor rule [11]. (see Appendix 3). A more detailed description of the selection procedures can be found in Appendix 2.

### Statistical analysis

A complete case analysis was performed since the percentage of missing values was lower than 10% [10, 18, 24]. Before building the model, underlying assumptions of linearity between independent continuous variables and the outcome and multicollinearity for the potential predictors were checked [3].

Two prognostic models were developed, one for the outcome at 6 months and one at 24 months, using a multivariable logistic regression analysis with Backward Wald Selection and a cut-off of *p* < 0.157 [11, 18]. The performance of the models was assessed by the explained variance, the calibration and the discriminative ability of the models [3, 11, 18]. The explained variance is determined by the Nagelkerke’s *R*^2^ statistic, with a larger *R*^2^ indicating that a larger proportion of the variance can be explained by the model. Calibration, also called goodness of fit, was assessed by the Hosmer and Lemeshow test and calibration slope of the calibration plots [3, 18]. A good model fit was established when the Hosmer and Lemeshow test was not significant. The calibration slope indicates an over-, smaller than 1, or underfitting, larger than 1, of the model. The discriminative ability of the models was determined by the Area under the Curve (AUC) [11, 18]. An AUC between 0.6 and 0.8 was considered acceptable and a value of 0.8 or higher represents good discriminative ability of the model [11]. All statistical analyses were performed using IBM SPSS, version 22 (IBM Corp, Armonk, NY, USA).

To correct for the optimism in the prognostic model the final model was internally validated using bootstrapping technique with 500 repetitions [3, 11] The statistical software R-studio version 1.2.1335 (R-studio Inc., Boston, MA, USA) was used for the internal validation,. The correction factor from the bootstrapping was applied to the regression coefficients and performance measures.

## Results

A total of 161 patients were allocated to physical therapy. Eight patients were excluded prior to data analysis because they attended less than six physical therapy sessions. At 6 months, 32 patients (20.9%) had undergone meniscal surgery. At 24 months, an additional 11 patients had undergone meniscal surgery, resulting in a total of 43 patients (28.1%). The baseline characteristics of both groups are presented in Table 1.

**Table 1:** Baseline characteristics per group for the models at 6 and 24 months

	Model at 6 months			Model at 24 months		
	No meniscal surgery	Meniscal surgery after PT	*p* value^*a*^	No meniscal surgery	Meniscal surgery after PT	*p *value^*a*^
**Demographics**	*N* = 121	*N* = 32		*N* = 110	*N* = 43	
Age in years	57.2 (6.8)	57.4 (7.0)	n.s.	57.7 (7.0)	56.4 (6.7)	n.s.
Women	63 (52.1%)	16 (50.0%)	n.s.	58 (52.7%)	21 (48.8%)	n.s.
Body Mass Index	27.0 ( 4.0)	27.6 (3.9)	n.s.	27.1 (4.1)	27.2 (3.7)	n.s.
Education level (score is 1–7)^b^	4.8 (1.8)	3.8 (1.7)	0.05	4.8 (1.8)	4.0 (1.8)	*0.02*
Smoking (yes)	16 (13.2%)	3 (9.4%)	n.s.	14 (12.7%)	5 (11.6%)	n.s.
**Patient reported outcomes**						
Pain during activities (NRS; 0–100)	56.1 (22.4)	67.9 (21.1)	0.02	56.1 (22.2)	64.9 (22.5)	n.s.
Knee function (IKDC; 0–100)	48.8 (14.1)	39.6 (13.6)	< 0.01	48.9 (14.2)	41.7 (14.2)	0.05
Physical health (Rand-36 PCS; 0–100)	38.7 (8.7)	36.2 (8.1)	n.s.	38.8 (8.7)	36.5 (8.4)	n.s.
Patient expectation						
No pain relieve within 6 months	11 (9.1%)	7 (21.9%)	n.s.	10 (9.1%)	8 (18.6%)	n.s.
Pain relieve within 6 months	110 (90.9%)	25 (78.1%)		100 (90.9)	35 (81.4%)	
**Imaging results**						
OA score on radiograph (KL classification)^d^			n.s.			n.s.
0: No OA	12 (9.9%)	2 (6.3%)		5 (4.5%)	0 (0%)	
1: Doubtful	55 (45.5%)	16 (50%)		37 (33%)	10 (21.7%)	
2: Minimal OA	45 (37.2%)	8 (25%)		35 (31.3%)	9 (19.6%)	
3: Moderate OA	2 (1.7%)	2 (6.3%)		6 (5.4%)	1 (2.2%)	
4: Severe OA^e^	0 (0%)	0 (0%)		0 (0%)	0 (0%)	
Affected meniscus^f^			n.s.			n.s.
Medial	93 (76.9%)	25 (78.1%)		86 (78.2%)	32 (74.4%)	
Lateral	19 (15.7%)	5 (15.6%)		17 (15.5%)	7 (16.3%)	
Both	9 (7.4%)	2 (6.3%)		7 (6.4%)	4 (9.3%)	

Data are presented as *n* (%) or mean (SD)

*NRS *numeric rating scale, higher score indicates more pain, *IKDC *International Knee Documentation Committee Subjective Knee Form, higher score indicates better knee function, *RAND-36 PCS *physical component score of the RAND-36 questionnaire, higher score indicates better physical health status, *OA *osteoarthritis, *KL *Kellgren–Lawrence classification of knee osteoarthritis, *n.s.* not significant

^a^Statistical differences between the surgery after PT group and no meniscal surgery group was assesses by an independent-sample *T* test for continues data, or a χ^2^ test for binary and categorical data. *p* values ≤ 0.05 were considered significant

^b^Education level measured according to the International Standard Classification of Education (ISCED) score ranges from 1 to 7 with a higher score indicating higher level of education

^c^Expectation of the pain score, 1 = pain will get severely worse and 6 = pain will be relieved completely

^d^Grade of knee osteoarthritis was assessed by X-ray using the Kellgren and Lawrence scale (K&L)

^e^Patients with a KL classification of 4 on the baseline X-ray were excluded from the trial

^f^Location of tear was assessed by magnetic resonance imaging

In the 28.1% of patients who underwent a meniscal surgery, 8 patients (18.6%) expected no relieve in pain (score 1–4) following physical therapy. In the patients who did not undergo surgery, 10 patients (9.1%) expected no relieve in pain following physical therapy.

### Multivariable regression analyses

A complete case analysis was performed for both models with 153 cases. The model at 6 months confirmed all three candidate predictors as significant prognostic predictors: patient-reported knee function, education level and general physical health. Patients with worse knee function at baseline, a lower level of education and better self-reported general physical health had a higher probability of undergoing meniscal surgery. The results of the multivariable regression analyses, model performance measures and internal validation are presented in Table 2. The explained variance of the model was 16%, indicating that the predicted outcome can be explained for 16% by the predictors. The Hosmer and Lemeshow test was 0.12 and the mean calibration 0.003, indicating a good model fit. However, the calibration plot displayed an overestimation of the predicted outcomes for the model with a calibration slope of  < 1. The discriminative ability of the model was adequate with an AUC of 0.73. Internal validation resulted in a correction factor for the initial model’s optimism of 0.90. The correction factor was applied to the regression coefficients and performance measures.

**Table 2:** Prognostic models for meniscal surgery after initial PT treatment at 6 and 24 months

Predictor	Beta^a^	Adusted beta^b^	OR (95% CI)	*p *value^c^
Model at 6 months				
Knee function^d^	−0.06	−0.05	0.94 (0.90–0.98)	0.01
Education level^e^	−0.25	−0.23	0.78 (0.62–0.99)	0.04
General physical health^f^	0.05	0.05	1.05 (0.98–1.13)	0.15
Model at 24 months				
Knee function^d^	–0.03	–0.03	0.97 (0.94–1.00)	0.03
Education level^e^	–0.17	–0.14	0.84 (0.69–1.03)	0.10

Model performance	*R*^2^	AUC	Mean calibration	H&L
Model at 6 months				
Initial model	0.16	0.73	0.003	0.12
After internal validation^b^	0.14	0.71		
Model at 24 months				
Initial model	0.11	0.68	0.001	n.s
After internal validation^b^	0.09	0.66		

*95% CI *95% confidence interval, *OR *odds ratio, *R*^2^ Nagelkerke’s *R*^2^, *AUC *area under the curve, *H&L *Hosmer and Lemeshow test, *n.s.* not significant

^a^Positive beta is indicative that a higher score results in a higher probability of undergoing a meniscal surgery; a negative coefficient indicates that this risk increased with lower score. Some multicollinearity between the predictors can explain apparent discrepancies with baseline (Table 1)

^b^Regression coefficients and performance measures for the model at 6 months were multiplied by the shrinkage factor of 0.90 retrieved from internal validation

Regression coefficients and performance measures for the model at 24 months were multiplied by the shrinkage factor of 0.82 retrieved from internal validation

^c^*p* values lower than 0.157 are considered significant

^d^Knee function measured with the International Knee Documentation Committee Subjective Knee Form (IKDC) score ranges from 0 to 100, a higher score indicates better knee function

^e^Education level measured according to the International Standard Classification of Education (ISCED) score ranges from 1 to 7 with a higher score indicating higher level of education

^f^General physical health measures with the RAND-36 Physical Component Score. Scores ranges rom 0–100, higher score indicates better health status

The model at 24 months confirmed that worse patient-reported knee function and lower level of education were prognostic factors for undergoing meniscal surgery. Patients with worse knee function at baseline, a lower level of education had a higher probability of undergoing meniscal surgery. The results of the multivariable regression analyses, model performance measures and internal validation are presented in Table 2. The explained variance of the model was 11%, indicating that the predicted outcome can be explained for 11% by the predictors. The Hosmer and Lemeshow test was 0.48 and the mean calibration 0.002, indicating a good model fit. However, the calibration plot displayed an overestimation of the predicted outcomes for the model with a calibration slope of  < 1. The discriminative ability of the model was adequate with an AUC of 0.68. Internal validation resulted in a correction factor for the initial model’s optimism of 0.82 for the regression coefficients and performance measures.

## Discussion

Two prognostic models were developed and internally validated to predict which patients will undergo meniscal surgery following physical therapy in patients with a degenerative meniscal tear. Patients who experienced a better general physical health status (for the 6 months model), and had worse knee function and lower education level (for both the 6 and 24 months model) were more likely to undergo meniscal surgery. However, both models showed a low explained variance and had an insufficient predictive accuracy. Therefore, external validation of these models is not useful since the models cannot be used in clinical practice.

Predicting treatment outcome for patients with a meniscal tear remains challenging. Recently, a study investigated the ability of orthopedic surgeons to predict the outcome of physical therapy and the outcome of surgery in patients over 45 years with a symptomatic meniscal tear [20]. Orthopedic surgeons received baseline characteristics of the patient including demographic information about employment, age and BMI, PROMs on pain, knee function and mechanical dysfunction, and MRI results on tear type and location, and radiograph information on level of knee osteoarthritis. Similar to the results of this study, they found that orthopedic surgeons were also unable to accurately predict which patient would benefit from physical therapy based on the baseline characteristics [20].

Multivariable prognostic prediction models have also been shown inaccurate in predicting the treatment outcome of initial meniscal surgery in a similar population [13]. The authors argued that treatment outcome cannot be accurately predicted in this population due to the combination of knee osteoarthritis and a meniscal tear, which is a common finding in middle aged and older patients [13]. Likewise, mild to moderate knee osteoarthritis was also found in our study in the majority of the patients [21]. This may have negatively impacted on the predictive ability of our models since patients might experience persistence of knee complaints due to overall degenerative knee pain instead of solely meniscal pain [13]. The current literature appears to report similar results as the current study which suggests that no subgroups can be identified who can benefit from surgery. The current study supports other literature that failed to identify subgroups of patients who can benefit from surgery [13]. The efficacy of physical therapy was not investigated in this study. However, given the absence of a clinically relevant benefit of surgery over conservative treatment [2, 4–7, 9, 12, 16, 17, 21, 25], and the lack of clear prognostic characteristics for treatment outcomes [13], clinicians should rely more on the current guidelines recommending physical therapy as the first-line treatment in patients with degenerative meniscal tears. [15, 19]

This study has some limitations. First, this study was not primary designed as a prognostic prediction model study. Using data collected within a RCT is suitable to develop prognostic models [11]. Nevertheless, it was a disadvantage that the variables, available for the development of the models, did not include some of the variables that were previously shown to be associated with the outcome. For instance, from the current literature the variable duration of symptoms was selected as prognostic factor [8]. However, duration of symptoms was not assessed in our study population and could therefore not be included in the model [23]. Second, the amount of candidate predictors was determined using the rule of 10 events per potential predictor. Although this is an accepted method and recommended in the TRIPOD statement, some researchers suggest that the rule of thumb is too simplistic to determine an adequate sample size for multivariable prognostic models with a binary outcome [14]. In our study the sample size and amount of events was fixed since data were used that were collected within the ESCAPE trial. Therefore the amount of candidate predictors was determined on our sample size, instead of vice a versa. Nevertheless, the results of this study meet the criteria, a shrinkage factor of ≥ 0.9 that represents a small optimism in predictor effect estimates and a small absolute difference of ≤ 0.05 in the model's initial and adjusted Nagelkerke’s *R*^2^, that Riley et al. [14] proposed for an adequate sample size. Last, the prescribed PT treatment was a standardized incremental exercise protocol.

With the current available evidence, it is impossible to identify which patient will require surgery following physical therapy. Instead, clinicians should recommend physical therapy as the first-line treatment for patients with degenerative meniscal tears, following the current guidelines [15, 19]

## Conclusion

With this study, the course of conservative treatment could not be predicted and patients who are likely to undergo meniscal surgery in the short (i.e., 6 months) and long term (i.e., 24 months) following physical therapy could not be identified. Therefore, these models should not be externally validated and not used in clinical practice. Future research should focus on identifying specific prognostic factors for treatment selection, surgery or physical therapy, in this population.

## Supplementary Information

Below is the link to the electronic supplementary material.

Supplementary file1 (DOCX 33 KB)
