# Supplementary material for: In patients eligible for meniscal surgery who first receive physical therapy, multivariable prognostic models cannot predict who will eventually undergo surgery
Source: Knee Surg Sports Traumatol Arthrosc. 2021 Feb 7;30(1):231–8. doi: 10.1007/s00167-021-06468-0 (PMC8800906; doi:10.1007/s00167-021-06468-0)
Supplement: Supplementary file 1 — Supplementary file1 (DOCX 33 KB) [file 167_2021_6468_MOESM1_ESM.docx]

**Appendix 1. Physical therapy exercise program.**

__________________________________________________________________________________

Time Exercises Repetitions or time

(week)

__________________________________________________________________________________

**0-8** Stationary cycling for warming up 15 min or longer and cooling down or cardiovasculair training *gradually increase intensity*

**0-8** Pully or dynaband, strapped around ankle uninjured side. 3x12 reps

Stand on injured side and keep balance, *each direction*

Step with uninjured side forward, backwards and sideways

**0-4** Calf raises on a leg press machine 3x12 reps

**0-8** Hamstrings: standing hip extension in a 3x12 reps
 “multi-hip” trainings device

**0-4** Keeping balance on a balance board, use both feet

**0-8** Climbing stair , walking, acceleration, running, 10 min

Jumping. *According to the patients activity level*

**5-8** Calf raises standing on one leg 3x12 reps

**1-8** Leg press, place feet high enough for the 3x12 reps
shinbones to become in a horizontal plane
and the knee starting at 110˚ flexion, unilateral

**5-8** Squats (according the needs of the patient) 3x12 reps

In which a knee flexion > 90° is not allowed

**5-8** Balance board on one foot 3 min

challenge with throwing a ball

**5-8** Elliptical machine for warming up 10 min or longer and cooling down or cardiovascular training

**The exercise program contained 16 supervised sessions during 8 weeks**

For all exercises is it important to keep the patient’s individual needs and limitations focused by using the ICF. The uninjured side is also less trained as usual and therefore both sides should be trained.
Besides training of the lower extremity, “core stability” training is of importance for good posture positioning and moving. The active rehabilitation program is designed around cardiovascular (circulation), coordination and balance, and closed chain strength exercises. Shearing forces in the knee are less using closed chain exercises compared to open chained exercises. The closed chain exercises activate both agonists and antagonists around the knee joint resulting in a direct rotatory movement and prevent in shearing forces seen by open chained exercises.

**Home exercise program**
In addition, a home exercise program was provided to all participants. It consisted of one leg standing during 60 seconds and a step-down exercise comprising 3, 9, 10 repetitions, twice a week.

**Appendix 2. Detail description of selecting candidate predictors.**

From an extensive list of baseline variables assessed within the ESCAPE trial we selected candidate predictors using a combination of three methods. First, we conducted a literature search to identify factors associated with the outcome for physical therapy in patients with a meniscal tear.
To identify studies on prognostic factors, a search strategy was developed with the assistance of an independent librarian. We searched the database of PubMed in March 2019. Two researchers (BLINDED) independently selected the studies that identified factors associated with treatment outcome in patients with meniscal tears. We included a study for our candidate predictor selection if one of the researchers selected the article based on the title and abstract containing information on predictive factors for the outcome of physical therapy for patients with a meniscal tear. Both researchers then read the full text article and excluded studies that reported on a different study population or that did not report any predictive factors for the treatment outcome of physical therapy. The selected studies were evaluated on the level of evidence by the Grading of Recommendations, Assessment, Development and Evaluations (GRADE). Finally, we identified all possible predictors from the selected studies that were also measured within the ESCAPE trial.

*Search strategy:*

Search (((("Meniscus"[Mesh] OR menisc*[tiab]) AND (tear*[tiab] OR injur*[tiab] OR lesion*[tiab] OR rupture*[tiab])) AND ("Conservative Treatment"[Mesh] OR "Rehabilitation"[Mesh] OR "Physical Therapy Modalities"[Mesh] OR "Physical Therapy Department, Hospital"[Mesh] OR "Exercise"[Mesh] OR "Exercise Movement Techniques"[Mesh] OR "Exercise Therapy"[Mesh] OR "Physical Therapy (Specialty)"[MeSH] OR rehabilitati*[tiab] OR physiotherap*[tiab] OR ((physical[tiab] OR conservative[tiab]) AND (therapy[tiab] OR therapies[tiab] OR activity[tiab] OR activities[tiab] OR treatment*[tiab] OR management*[tiab])) OR exercis*[tiab] OR training*[tiab]) AND ("Epidemiologic Studies"[Mesh] OR cohort[tiab] OR (case[tiab] AND (control[tiab] OR controll*[tiab] OR comparison[tiab] OR referent[tiab])) OR risk[tiab] OR causation[tiab] OR causal[tiab] OR "odds ratio"[tiab] OR etiol*[tiab] OR aetiol*[tiab] OR "natural history"[tiab] OR predict*[tiab] OR prognos*[tiab] OR outcome[tiab] OR course[tiab] OR retrospect*[tiab]))) AND ((("Patient Satisfaction"[Mesh] OR patient satisfaction[tiab] OR satisfaction[tiab])) OR (cross over) OR (IKDC[tiab] OR outcome*[tiab] OR KOOS[tiab] OR WOMAC[tiab]))

Second, we sent an electronic survey to the orthopaedic surgeons (n=24) and physical therapists (n=22) who were involved in the ESCAPE trial. We gave the respondents 4 weeks, in which we sent 3 reminders, to respond to our survey. A total of 12 orthopaedic surgeons (50%) and 5 physical therapists (23%) completed the survey. Additionally, 10 patients from the ESCAPE trial completed the survey by phone with the assistance of a researcher (BLINDED) to clarify medical jargon.

The survey contained all baseline variables measured in the ESCAPE trial. The respondents received written instructions to select all clinically relevant prognostic factors for the treatment outcome, in their opinion. We then ranked the list of predictors by percentage and selected the 10 most chosen prognostic factors.

Third, we conducted a univariate logistic regression analysis preselection. We considered a predictor as significant with a p-value of 0.05 or lower. The principle researchers of this study (BLINDED) made a final selection of 12 potential predictors. None of the studies that resulted from our search identified a clear association between the type of tear and the outcome of a patient undergoing physical therapy. Despite the absence of the type of tear and affected meniscus in the literature search and the survey, these predictors were deemed clinically relevant by the expert panel. For these two variables (type of tear and affected meniscus) we therefore performed a univariable logistic regression analysis to rule out the possibility of missing potentially important predictors.

**Appendix 3. Table of candidate predictors and selection procedure.**

| Candidate Predictor | Literature^a^ | Expert panel survey^b^ | Univariate analysis^c^ | Principle researchers’ ranking of predictors^d^ |
| --- | --- | --- | --- | --- |
| Knee function* | Yes^2-4^ | 56% | P<0.01 | 1 |
| Education level* | No | 56% | P<0.01 | 2 |
| General physical health* | Yes^5^ | 42% | P=0.09 | 3 |
| Body Mass Index** | Yes^6^ | 85% | P=0.20 | 4 |
| Pain during activities | Yes^6^ | 78% | P<0.01 | 5 |
| Knee arthrosis | Yes^7^ | 46% | P<0.68 | 6 |
| Knee effusion | No | 63% | P=0.57 | 7 |
| Age | No | 56% | P=0.96 | 8 |
| Patient’s expectation | No | - | P=0.09 | 9 |
| Mechanical complaints  Meniscus affected:  Lateral  Medial  Both  Meniscus type (ISAKOS):  Longitudinal-vertical  Horizontal  Radial  Vertical flap  Complex degenerative  Not able to classify | No  No  No | -  -  - | P=0.36   P=0.97  P=0.82  P=0.86    P=0.80 P=0.80 P=0.80  P=0.80 P=0.80  P=0.77 | 10  11  12 |
| Abbreviations: ISAKOS= the International Society of Arthroscopy, Knee Surgery and Orthopaedic Sports * These candidate predictors were included in the initial multivariable prognostic model at 6 months  ** BMI was added to the selected candidate predictors in the initial multivariable prognostic model at 24 months  ^a^ Identification of candidate predictors from the current literature on factors for prognosis of physical therapy in patients with a meniscal tear.  ^b^ Identification of relevant prognostic factors by the expert panel of orthopaedic surgeons, physical therapists and patients, thru an online survey. The numbers indicate the percentages of the expert panel that selected the variable as relevant predictor for the prognosis of physical therapy in patients with a meniscal tear.  ^c^ Univariate logistic regression analysis was conducted to included additional prognostic factors. P ≤ 0.05 was considered significant.  ^d^ The principle researchers of this study made a final ranking of 10 potential predictors based on whether the prognostic factor could be influenced by physical therapy, clinical relevance, the applicability in the clinical setting and aiming to cover all components of the biopsychosocial model. | | | | |

**References**

1. Engel GL. (1977) The need for a new medical model: a challenge for biomedicine. *Science*. 196:7. doi: 10.1126/science.847460

2. Rathleff CR, Cavallius C, Jensen HP, et al. (2015) Successful conservative treatment of patients with MRI-verified meniscal lesions. *Knee Surg Sports Traumatol Arthrosc*. 23(1):178-83. doi: 10.1007/s00167-013-2494-z [published Online First: 2013/04/12]

3. Stensrud S, Roos EM, Risberg MA. (2013) A 12-week exercise therapy program in middle-aged patients with degenerative meniscus tears: a case series with 1-year follow-up. *J Orthop Sports Phys Ther*. 42(11):919-31. doi: 10.2519/jospt.2012.4165 [published Online First: 2012/09/11]

4. Katz JN, Wright J, Spindler KP, et al. (2016) Predictors and Outcomes of Crossover to Surgery from Physical Therapy for Meniscal Tear and Osteoarthritis: A Randomized Trial Comparing Physical Therapy and Surgery. *J Bone Joint Surg Am*. 98(22):1890-96. doi: 10.2106/JBJS.15.01466 [published Online First: 2016/11/18]

5. Kudo M, Watanabe K, Otsubo H, et al. (2013) Analysis of effectiveness of therapeutic exercise for knee osteoarthritis and possible factors affecting outcome. *J Orthop Sci*. 18(6):932-9. doi: 10.1007/s00776-013-0443-9 [published Online First: 2013/10/03]

6. van de Graaf VA, Noorduyn JCA, Willigenburg NW, et al. (2018) Effect of Early Surgery vs Physical Therapy on Knee Function Among Patients With Nonobstructive Meniscal Tears: The ESCAPE Randomized Clinical Trial. *JAMA*. 320(13):1328-37. doi: 10.1001/jama.2018.13308 [published Online First: 2018/10/05]

7. MacFarlane LA, Yang H, Collins JE, et al. (2017) Influence of Baseline Magnetic Resonance Imaging Features on Outcome of Arthroscopic Meniscectomy and Physical Therapy Treatment of Meniscal Tears in Osteoarthritis. *Am J Sports Med*. 47(3):612-19. doi: 10.1177/0363546518819444 [published Online First: 2019/01/18]
